# Clinical characteristics and survival outcomes of malignant struma ovarii confined to the ovary

**DOI:** 10.1186/s12885-021-08118-7

**Published:** 2021-04-09

**Authors:** Sijian Li, Tengyu Yang, Yang Xiang, Xiaoyan Li, Limeng Zhang, Shan Deng

**Affiliations:** 1 Department of Obstetrics and Gynecology, Peking Union Medical College Hospital, Chinese Academy of Medical Sciences, Peking Union Medical College, Beijing, People’s Republic of China; 2 Department of Otolaryngology, Peking Union Medical College Hospital, Chinese Academy of Medical Sciences, Peking Union Medical College, Beijing, People’s Republic of China

**Keywords:** Malignant struma ovarii, Thyroid carcinoma, Surgery, Adjuvant therapy, Prognosis

## Abstract

**Background:**

Malignant struma ovarii (MSO) is a unique type of ovarian malignancy that data on the survival outcome is limited and management strategy remains controversial due to its extreme rarity.

**Methods:**

To investigate the clinical characteristics and treatment options in patients with MSO confined to the ovary, while also evaluating the recurrent-free survival (RFS) and overall survival (OS) rate in this population, a retrospective study was conducted. One hundred twenty-five cases of MSO confined to the ovary were enrolled and their clinical characteristics, treatment strategies, and results of follow-up were analyzed. OS and RFS were assessed by Kaplan-Meier analyses and Cox regression models.

**Results:**

The most common pathological subtype in this cohort was papillary carcinoma (44.8%). Other reported subtypes, in order of prevalence, were follicular variant of papillary carcinoma, follicular carcinoma, and mixed follicular-papillary carcinoma. Surgical treatment options varied in this cohort that 8.0% of the patients received ovarian cystectomy, 33.6% underwent unilateral salpingo-oophorectomy (USO), 5.6% received bilateral salpingo-oophorectomy (BSO), 21.6% received total abdominal hysterectomy with BSO (TAH/BSO), and 17.6% were treated with debulking surgery; 20.0% of them received radioiodine therapy (RAI). Twenty-seven patients experienced recurrence with a median RFS of 14.0 years (95% confidence interval [CI], 9.5–18.5). The 5-year and 10-year recurrent rate were 27.1, 35.2%, respectively. Eight patients died during follow-up, with five attributed to MSO; the 5-year, 10-year, and 20-year OS rate was 95.3, 88.7 and 88.7%, respectively. However, the univariate and multivariate Cox regression showed no potential risk factor for RFS and OS.

**Conclusion:**

Patients with MSO confined to the ovary had an excellent survival outcome, despite varied treatment strategies, and the recurrent rate was relatively high. We recommend USO as the preferred surgical option in this population since more aggressive surgery does not improve outcomes and the benefits of RAI are uncertain.

**Supplementary Information:**

The online version contains supplementary material available at 10.1186/s12885-021-08118-7.

## Background

Struma ovarii is a form of ovarian teratoma defined by the presence of thyroid tissue comprising more than 50% of the tumor [1]. Approximately 2–5% of all ovarian teratomas and 0.5–1% of all ovarian tumors can be classified as struma ovarii [2–4], and malignant struma ovarii (MSO) accounts for approximately 5–10% of struma ovarii that can be histologically identified as differentiated thyroid carcinoma [5, 6]. MSO occurs most commonly in women in their 30s and 40s with widely varied clinical manifestations [7, 8]. There are fewer than 200 cases reported in literature so far [7] and due to its rarity, there is currently no consensus on management of MSO, and further evidence is needed on the factors affecting prognosis to reach this consensus. Evidence on and the benefits of different surgical options mainly originate from case reports.

DeSimone et al. and Shrimali et al. both suggested that MSO should be treated with a combination of local surgery, a total thyroidectomy, and radioiodine therapy (RAI) for both patients with and without extra-ovarian metastasis [9, 10]. For women of childbearing age, a more conservative management option is certainly practical, while complete staging surgery should be performed in patients who are not concerned with fertility preservation [9]. However, McGill et al. and Marti et al. advocated that RAI should be reserved only for patients who have evidence of metastasis [11, 12]. They also found the recurrent rate was as low as 7.5% in 25-year-olds with well-differentiated thyroid cancer within an ovary, therefore pelvic surgery alone may be adequate in this population [12]. Nonetheless, other studies reported a much higher recurrent rate, ranging from 22 to 35% [9, 13]. While the survival rate in patients with MSO is excellent regardless of management strategy employed [8, 14], the specific pelvic surgical option that should be prioritized and the benefits of aggressive surgery are still uncertain. Moreover, none of these studies emphasized the overall survival (OS) rate in patients with MSO within the ovary with varied differentiated degrees, and the factors associated with recurrent-free survival (RFS) and OS have not been well defined.

Here we aimed to present the clinical and pathological characteristics and treatment options for patients with MSO confined to the ovary, while also investigating the RFS and OS rate, as wells as factors that affect both rates in this population. We included five cases of MSO confined to the ovary from our hospital and comprehensively reviewed another 120 cases documented in literature from MEDLINE from the last 80 years.

## Methods

This retrospective study was approved by the Ethics Committee of Peking Union Medical College Hospital. A total of five cases of MSO within the ovary diagnosed in the past 20 years at the Peking Union Medical College Hospital were identified. Their clinical data, including demographics, clinical characteristics, treatment strategies and results of follow-up, were described and analyzed. A similar strategy for systematic literature review described in previous study [15] was conducted that English literature published from 1940 to 2020 were reviewed in PubMed (https://www.pubmed.gov) using the following keywords: “malignant struma ovarii”; “metastatic malignant struma ovarii”; “malignant ovarian teratoma”; “thyroid carcinoma arising in struma ovarii”; “struma ovarii”. Moreover, we evaluated references that cited by these articles. Patients with MSO confined to the ovary were included in our study. The exclusion criteria included benign struma ovarii, MSO with extra-ovarian spread at initial diagnosis, MSO found in autopsy, and MSO without clinical characteristics and outcomes. A total of 120 cases of MSO were included after screening (The detailed inclusion process is shown in Supplementary Fig. S1). A database including demographic and clinical characteristics from these 120 cases and the five cases from our hospital was established. The following characteristics were analyzed to identify independent factors that might predict disease prognosis: age at diagnosis, where the age of 55 years was selected as the cut-point according to the American Joint Committee on Cancer (AJCC) staging system for well-differentiated thyroid cancers [16]; ascites (with or without); tumor size, where the cut-point was examined in different values according to a previous study which evaluated risk factors for prognosis [2, 14, 17]; pathological subtypes (follicular carcinoma or not); surgical options, which included ovarian cystectomy, unilateral salpingo-oophorectomy (USO), bilateral salpingo-oophorectomy (BSO), total abdominal hysterectomy with BSO (TAH/BSO); debulking surgery; adjuvant therapy (with or without RAI); recurrence (yes or no). Recurrence free survival (RFS) was defined as the date from initial surgical intervention to confirmed tumor recurrence or metastasis. Overall survival (OS) was defined as the date from initial surgical intervention to MSO related death or final follow up.

### Statistical analysis

Continuous variables were presented by means and standard deviations (range). Discrete variables were described by counts (percentage). Survival analyses were performed using Kaplan-Meier analysis. The comparison of survival rates among the groups was performed by two-tailed log-rank test. Univariable analyses for RFS and OS were performed to select variables for further evaluation in multivariable models. Factors with *p*-values < 0.2 were included to multivariate analysis using the Cox regression model to identify independent prognostic factors. We set the statistically significant level at two-sided *p* value < 0.05. All the statistical analyses were performed by SPSS (Version 21.0; SPSS Inc.; Chicago, IL, USA) or GraphPad Prism (Version 8.0; GraphPad Software Inc., San Diego, CA, USA) software.

## Results

### Results of five cases in Peking union medical college hospital

#### Demographic data and clinical characteristics

The mean age of the five patients was 52.4 years, with a median age of 44 years (range: 42–78). Elevated serum CA 125 levels were noted in two cases (40%) but no patients showed hyperthyroidism. All patients underwent surgery that resulted in the accidental findings of pelvic masses, which were suspected to be benign teratomas and were diagnosed after surgery. One patient (case 1) coexisted with metastasis of renal clear cell carcinoma. None of them showed discomfort before admission (Table 1).

**Table 1:** Demographic and clinical characteristics of the five patients in our hospital

No.	Age (y)	Dysthyroidism; Elevated CA 125	Pathology	Surgery	Adjuvant therapy	Recurrence	Result of follow-up
1	78	N/N	FVPTC	Partial pancreatectomy (metastasis of clear cell carcinoma of kidney), splenectomy; USO (LSO)	N	N	NED at 40 m (Normal thyroid gland on US/WBS)
2	42	N/N	PTC	USO (LSO)	N	N	NED at 5y (Normal thyroid gland on US)
3	42	N/N	PTC	USO (LSO)	Chemotherapy (TC for 3 cycles)	N	NED at 3y (Normal thyroid gland on PET)
4	56	N/Y	PTC	USO, Debulking (TAH + RSO + omentectomy + LN)	Chemotherapy (TC for 6 cycles)	N	NED at 70 m (Normal thyroid gland on US)
5	44	N/Y	PTC	USO (LSO)	TT	N	NED at 6 m (TG undetectable); synchronous local primary PTC in neck

*Abbreviations*: *FVPTC* follicular variant papillary thyroid carcinoma, *PTC* papillary thyroid carcinoma, *USO* unilateral salpingo-oophorectomy, *L/RSO* left/right salpingo-oophorectomy, *BSO* bilateral salpingo-oophorectomy, *TAH* total abdominal hysterectomy, *LN* lymph nodes resection, *TC* taxol plus carboplatin, *TT* total thyroidectomy, *US* ultrasonography, *WBS* whole body scan, *NED* no evidence of disease

#### Surgical intervention, pathology, and adjuvant therapy

Four patients received USO. One patient (case 5) was initially treated with an ovarian cystectomy, sequentially followed by USO after diagnosis of MSO. The remaining patients were diagnosed with MSO by USO and received debulking surgery. Pathological examination showed that papillary carcinoma was the most common subtype (4/5, 80%), with one case of follicular variant papillary carcinoma. Two patients were treated with chemotherapy (carboplatin plus paclitaxel) postoperatively.

**Fig. 1:**
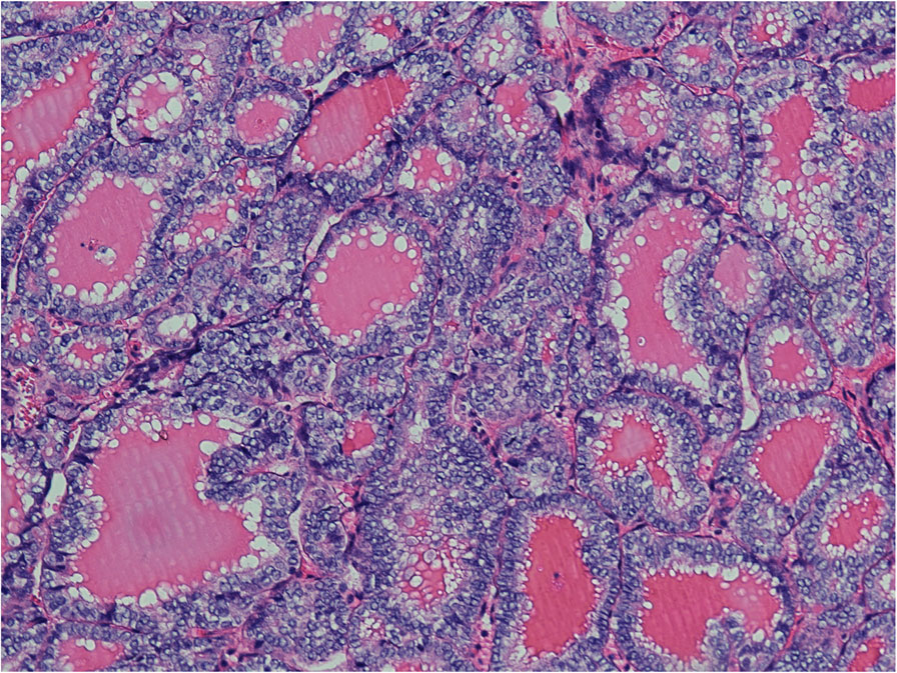
Typical pathological features of papillary carcinoma arising in struma ovarii (case 5, haematoxylin-eosin staining, 100X)

**Fig. 2:**
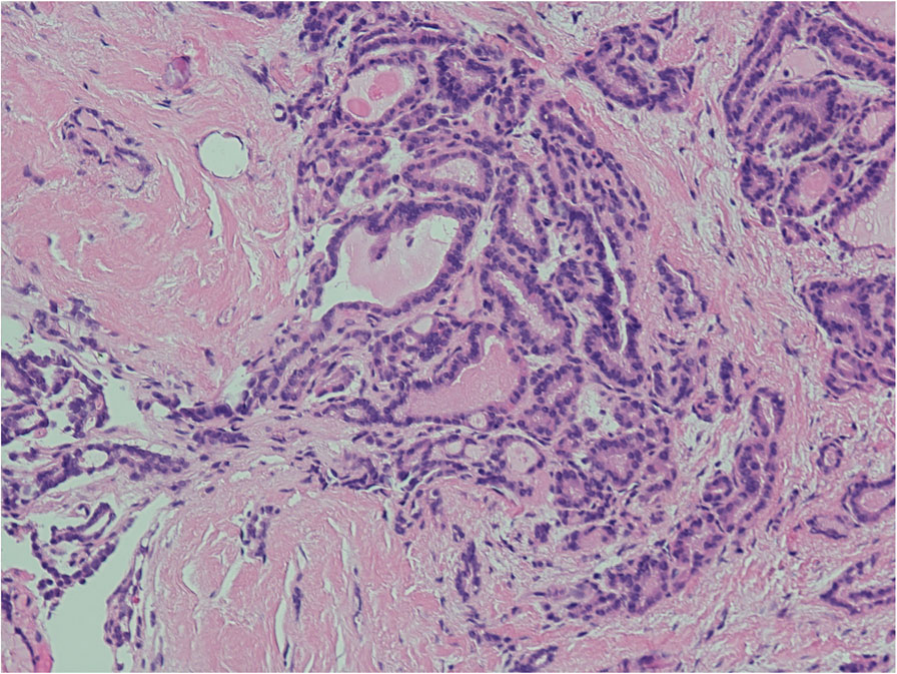
Typical pathological features of primary papillary carcinoma in neck coexisted with MSO (case 5, haematoxylin-eosin staining, 100X)

After being treated for ovarian tumors, thyroid cancer screening was conducted and four patients’ results were determined negatively by imaging; these patients did not receive further treatment. Only one patient was suspected of malignant nodules in the neck and received a total thyroidectomy; pathological results revealed primary papillary thyroid carcinoma without extra-thyroid spread (Fig. 1 and Fig. 2). No RAI was performed.

#### Patient follow-up

All patients lived without evidence of the disease during a median follow-up of 40 months (range 5–70) and none experienced recurrence.

### Database of 125 cases

A total of 125 patients with a median age of 46.0 years (range: 11 to 80) were included in the retrospective analysis (details can be found in the supporting information). Information on CA-125 was available in 33 patients and 12 (36.4%) had elevated levels of CA-125. In 108 recorded cases, 19 (17.6%) presented with ascites (database can be found in Additional file 1.).

**Fig. 3:**
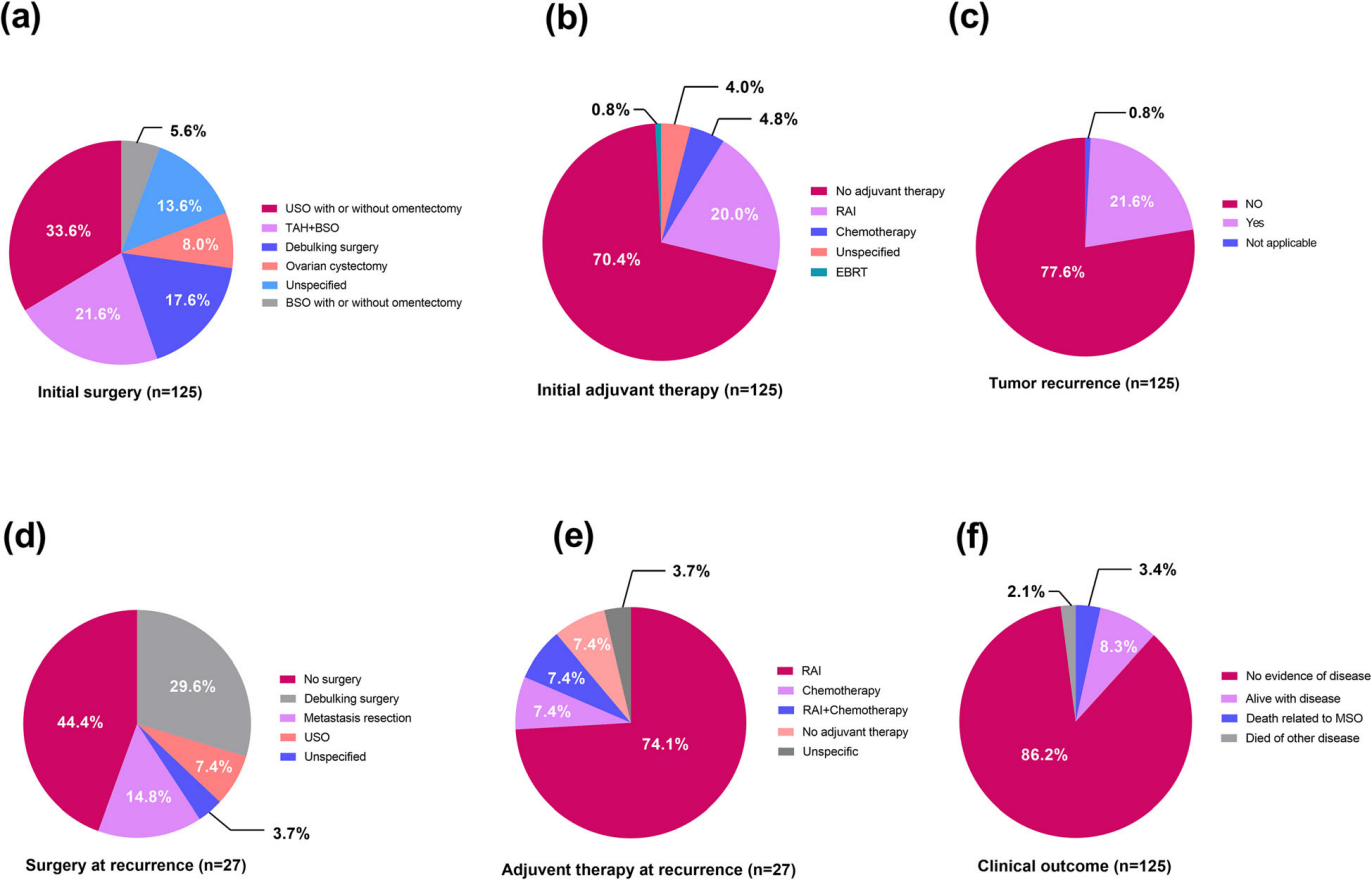
Treatment and clinical outcomes of MSO within ovary. **a** Surgical options, **b** adjuvant therapy, **c** tumor recurrence, **d** surgery at recurrence, **e** adjuvant therapy at recurrence and **f** clinical outcome

For the 73 cases with available data on tumor size, the mean tumor diameter was 7.7 ± 3.6 (range: 0.5 to 18.0) cm. Papillary carcinoma (44.8%) was the most prevalent pathological subtype. Other reported subtypes, in order of prevalence, were follicular variant of papillary carcinoma, follicular carcinoma, and mixed follicular-papillary carcinoma, and four cases were classified as poorly-differentiated thyroid carcinoma. All lesions were initially confined to a unilateral ovary, except for nine patients (7.2%) who coexisted with local primary thyroid cancer (Table 2). For the initial surgical option, a total of 42 patients (33.6%) underwent USO (with or without omentectomy), followed by TAH + BSO (21.6%) and debulking surgery (17.6%). Only 8.0 and 5.6% of cases received ovarian cystectomies or BSO (with or without omentectomy), respectively. Surgical approaches in the rest 17 cases (13.6%) were unspecified. Most cases (70.4%) did not receive any postoperative adjuvant treatment, while 20.0% were administered RAI and 4.8% received chemotherapy. Only one case received external beam radiotherapy (Fig. 3).

**Table 2:** Clinical characteristics of patients with MSO confined to the ovary

Patients’ characteristics	*N*	%	Recurrence	*N* = 124
Age (y)	125		Yes	27 (21.8%)
Mean 46.3 ± 14.2			Mean/Median time (y)	5.6/3.0
Median 46.0 (Range 11–80)			NA	1
Elevated CA 125	*N* = 33		**Sites of Recurrence**	***N*** **= 27**
Yes	12	36.4%	Peritoneum	12
Time of follow-up (y)	*N* = 125		Liver	8
Mean 5.12			Lung	7
Median 2.75 (Range 0.08–41)			Bone	5
Pathology	*N* = 125		Lymph nodes	4
PTC	56	44.8%	Diaphragm	4
FVPTC	39	31.2%	Bowel	4
FTC	23	18.4%	Omentum	2
Mixed FTC + PTC	3	2.4%	Fallopian tube	2
poorly differentiated TC	4	3.2%	Contralateral ovary	2
Synchronous primary thyroid carcinoma	*N* = 125		Bladder	1
Yes	9	7.2%	Mediastinum	1
Lymph nodes examination	*N* = 19		Uterus	1
Positive	0		Spleen	1
Mass size (cm) 7.7 ± 3.6 (0.5–18.0)	*N* = 73		Adrenal	1

*Abbreviations*: *MSO* malignant struma ovarii, *TC* thyroid carcinoma, *PTC* papillary thyroid carcinoma, *FTC* follicular thyroid carcinoma, *FVPTC* follicular variant of papillary thyroid carcinoma, *NA* not applicable

**Fig. 4:**
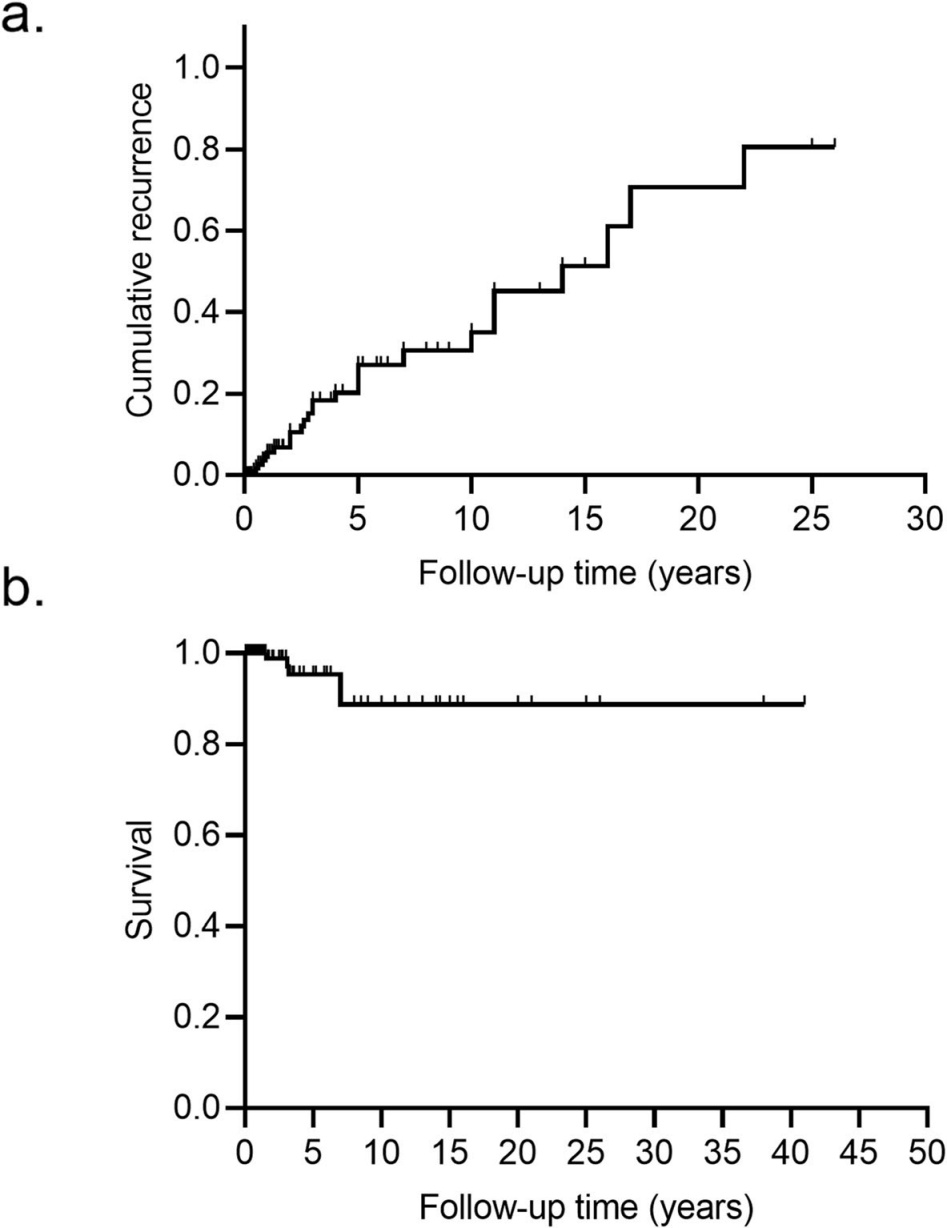
Survival curves in patients with MSO confined to ovary. **a** Overall survival of all patients enrolled in this study. **b** The cumulative recurrent rate was 27.1% at 5-year and 35.2% at 10-year in our study (*n* = 124)

Out of all the patients, 27 (21.8%) suffered from confirmed tumor recurrence and one patient did not achieve remission after initial treatment. The 5-year and 10-year RFS rates were 27.1 and 35.2% (Fig. 4a), respectively. The median recurrence duration was 14.0 (95%CI 9.5–18.5) years and most cases developed distant metastasis. The peritoneum was the most commonly involved site, followed by the liver, lung, and bone, in descending order. Other locations, such as lymph nodes, diaphragm, bowel, omentum, contralateral ovary, and fallopian tubes were less common. Uterus, bladder, spleen, adrenal gland, and mediastinum occasionally would be susceptible to tumor infiltration in cases of metastasis. Nearly half of the cases of recurrence did not receive further surgery, while the rest of them were mainly assigned debulking surgery. Of the patients that did not receive further surgery, 88.9% were treated with adjuvant therapy; 91.7% underwent RAI and 16.7% received chemotherapy. Adjuvant therapy was unspecified in one patient. (Potential prognostic factors for RFS were listed in Additional file 2.) Univariate analysis revealed that follicular carcinoma subtype and a lack of RAI were both likely to be associated with tumor recurrence. Further Cox proportional hazard analyses failed to identify any factors with statistical significance.

At the final follow-up, 105 (84.0%) cases achieved no evidence of disease (NED) status, 12 (9.6%) cases were alive with the disease (AWD), five (4.0%) cases died of the disease (DOD), and three (2.4%) died of other diseases. There was a wide range in the length of follow-up among the reported cases. The 5-year, 10-year, and 20-year OS rates were 95.3, 88.7, and 88.7%, respectively, with a mean OS of 37.0 years (95%CI 33.4–40.5) (Fig. 4b). (Potential prognostic factors affecting OS were listed in Additional file 3.) Univariant analysis using the Kaplan–Meier method showed that statistical differences in OS were associated with tumor recurrence (*p* = 0.008) and ascites at initial presentation (*p* = 0.01). These factors did not survive in further multivariable analyses.

## Discussion

Our study presented one of the largest cohorts concerning the management of disease in patients with MSO confined to the ovary and was the first to assess factors associated with RFS and OS in this population. The prognosis of MSO within the ovary was explored for varied treatment options, and we did not identify any statistically significant factors to predict RFS and OS. Based on the rarity and promising prognoses of these tumors and their lack of identified prognostic factors, an individualized and less aggressive treatment strategy is recommended.

Overall survival outcome in patients with MSO has been previously well demonstrated in two large cohort studies [8, 14]. In 2009, Robboy et al. reported that the OS rates of patients with struma ovarii with malignant behavior were 89 and 84% at the 10-year and 25-year time point, respectively [14]. However, 58 (66%) of their cases were follicular adenomatous or benign tumors; only 27 cases were strictly histologically consistent with MSO, which may have biased the true OS rate in patients with MSO. Goffredo et al. in 2015 published another study on 68 patients and revealed OS rates of 96.7, 94.3, and 84.9% at the 5-year, 10-year, and 20-year mark, respectively [8]. Their study was the first to present a more accurate OS rate in patients with MSO, but 20% of their cases were MSO with local or distant metastasis. Our current study provided a larger sample size and was restricted to those without extra-ovary spread at the initial diagnosis, and demonstrated a similarly excellent OS.

Overall recurrent rate in our research was 21.8%, with a cumulative recurrent rate of 27.1% at the 5-year and 35.2% at the 10-year mark, which was much higher than that of the 7.5% in the 25-year mark from Marti et al. [12]. However, it was compatible with the study reported by DeSimone et al., which had an overall recurrent rate of 35% in 24 patients [9], and Jean et al., which had an overall recurrent rate of 22% in 59 patients [13]. This may be attributed to the fact that Marti et al. restricted their selected patients to well-differentiated thyroid cancer. Meanwhile, we excluded more than 30 patients who lived with no evidence of disease recurrence but lacked individual detailed follow-up data in the literature [1, 14, 18–21]. This partly overestimated the recurrent rate. Although we reported a relatively higher recurrence rate, the cumulative recurrent rate was much lower than epithelial ovarian cancer [22]. Furthermore, an interesting result was that tumor recurrence was not a risk factor for OS, which was not consistent with common ovarian cancer [23]. This could be explained by the fact that the impact of MSO is generally milder and not lethal, as well as by its positive response to adjuvant therapy after recurrence. This reminds us that the treatment strategy of MSO needs to find a balance between pursuing radical treatment and improving long-term quality of life.

In our study, we also noted there was no potential risk factor for RFS and OS in patients with MSO confined to the ovary. Patients’ age at diagnosis, tumor size, presence of ascites, surgical options, pathological subtypes, RAI, and chemotherapy failed to predict either RFS or OS. Age over 55 years old was a strong predictor of relapse [24] and disease-specific death [16] in well-differentiated thyroid carcinoma. It is thought that older patients had a worse response to therapy (i.e. RAI) [25] and were not amenable to an aggressive therapeutic approach after the initial treatment [26]. However, all of our patients were diagnosed as MSO within the ovary at initial presentation and only 20.0% were initially administered RAI. A large cohort that includes MSO both with and without metastasis is needed to evaluate whether age at diagnosis can predict prognosis.

Several studies have investigated the risk factors for poor prognosis. For example, tumor size varying from 2 to 12 cm was proposed to predict an adverse clinical course or help classify “low-risk” and “high risk” patients [2, 14, 17]. Robboy et al. [14] suggested that a size of the strumal component ≥6 cm was related to disease recurrence while Shaco-Levy et al. [17] proposed that an overall size ≥10 cm and a strumal component ≥80% were associated with rapid disease progression and death. In this study, univariate analysis revealed that tumor size failed to predict either RFS or OS. However, most literature only provided the size of the struma instead of thyroid carcinoma because the carcinoma component is usually multifocal and blends together with the struma. It might be hypothesized that the size of the carcinoma component, rather than the strumal component, might affect the prognosis in patients with MSO, but further relevant research is needed.

Ascites more than one liter was also defined as a feature that can predict an adverse clinical course in previous studies [14, 17]. In our study, multivariate analysis showed that ascites was likely to predict OS (*p* = 0.054). This may be explained by that patient presented with ascites may in a more advanced tumor stage, while a more advanced stage of thyroid carcinoma or typical epithelial ovarian carcinoma both predict a poor prognostic [27, 28]. However, patients with benign struma ovarii can also present with ascites, such as in patients with pseudo-Meigs’ syndrome [29]. A previous study also suggested that histologic features used to predict clinical outcomes in thyroid tumors were not applicable to MSO [30]. We did not include these factors because most of the cases in our study had no detailed information about such histologic features. In addition, different subtypes of MSO showed similar RFS and OS in our study, which was not compatible with the previous study that found that recurrence of papillary carcinoma occurred earlier [14]. The lack of correlation between morphology and clinical outcome in MSO is striking, making the behavior of these tumors particularly unpredictable and the decision of treatment course more uncertain. Interpretation of the excellent prognosis of MSO may help to optimize disease management in this population.

Currently, the surgical options reported in the literature include an ovarian cystectomy, USO, BSO, TAH/BSO, and debulking surgery. However, the priority of these different surgical approaches has not been well evaluated in large cohorts, and evidence mainly relies on case reports. Some researchers argue for comprehensive staging surgery in postmenopausal patients or those who do not need to preserve fertility, otherwise USO should be preferred [9, 13]. We found that while both the RFS and OS rates were promising, there was no specific surgical option that could promise a more favorable prognosis. Ovarian cystectomies may cause the intraoperative rupture of tumor cysts, which may inevitably lead to the dissemination of tumor cells. This was not compatible with the tumor-free principle. Since nearly half of our patients were younger than 45 years old, surgical resection exceeding USO, especially debulking surgery, can increase trauma and blood loss and impact long-term quality of life. In reproductive women affected with MSO confined to the ovary, we strongly recommend USO as the preferred surgery. In perimenopausal or postmenopausal women, USO or BSO is more advisable for their feasibility prior to TAH/BSO and debulking surgery, regardless of their similar outcomes.

Whether RAI should be performed postoperatively has always been argued over [2, 9–13]. Most authors advocated for routine RAI to lower the recurrent rate after primary surgical resection of tumors [9, 10, 13]. However, Marti et al. found that pelvic surgery alone may be sufficient in patients without extra-ovarian metastasis [12]. Two large studies from Marti et al. and McGill suggested RAI should be reserved for cases with evidence of metastasis [11, 12]. Research published by Goffredo et al. found that only 9.2% of the 68 patients received RAI, but their survival outcomes were excellent [8]. In our study, the overall recurrent rate was significantly lower in patients treated with RAI than in those who received no RAI (3.8% vs. 26.1%, *P* = 0.014), yet its efficacy in improving RFS was not proven in univariate and multivariate analysis. Nonetheless, total thyroidectomies must be conducted before RAI, leading to a need for lifelong thyroxine supplements. Moreover, in premenopausal women, especially reproductive aged women, the impact of RAI on ovarian function cannot be neglected. Previous research demonstrated that RAI would lead to decreased ovarian function; the impact was more obvious in patients receiving multiple RAI therapies and in those older than 35 years [31]. Furthermore, ovarian function in patients with MSO has already been impaired by surgery. Knowing that the benefits of RAI on lowering recurrent rate is uncertain and it does not optimize OS, the use of RAI in patients with MSO confined to the ovary remains controversial. Physicians and patients must have clear communication before RAI and an individualized therapeutic plan taking into consideration patients’ personal intentions might be more practical.

Molecular profiling has been shown to impact clinical outcomes and help assess risk stratification of thyroid cancer. For example, BRAF^V600E^ mutation is associated with aggressive histologic features and metastases, and coexistent BRAF^V600E^ and telomerase reverse transcriptase (TERT) promoter mutations have a synergistic effect on increasing the risk of recurrence [32]. Published researches had also identified different somatic mutations in malignant struma ovarii, mainly in BRAF and RAS, as well as RET/PTC rearrangement [33–36]. Although MSO is histologically and genetically similar to primary thyroid carcinoma, currently no study has reported a molecular pattern that can predict more aggressive behavior in MSO. The reason may be that BRAF or RAS mutations and TERT promoter mutations were detected alone in 53 MSO cases with available data of molecular profile [35]. However, the fatal forms of non-ATC are generally PTC variants harboring BRAF or RAS mutations plus other genomic alterations such as TERT promoter [37]. Therefore, data were insufficient to evaluate the impact of molecular profiles on RFS or OS in patients with MSO. These researches indicated the potential significance of molecular features in guiding postoperative treatment, and further study should be conducted.

The follow-up strategy of patients with MSO confined to the ovary has not yet been well established. Serum thyroglobulin (TG) concomitant assessment of serum TG antibody (TGAb) and imaging should be the mainstays of MSO follow-up. Prophylactic total thyroidectomy to exclude a primary thyroid carcinoma and potentiate RAI therapy has been recommended, which allow for TG monitoring of possible metastases, remained mass or recurrence [9, 10]. However, our study revealed that total thyroidectomy followed by RAI is not mandatory for patients with MSO confined to the ovary. We recommend that all patients with MSO should have serum TG and TGAb assessments every 6–12 months refer to the guideline of thyroid cancer [38]. Meanwhile, the follow-up schedule may be individualized depends on the disease condition and initial treatment efficacy. We recommend that pelvic MRI can be performed every 1–2 years to exclude recurrent disease. Other imaging studies (WBS, FDG–PET, ultrasonography, and CT) should be ordered if locoregional or distant recurrences are presented, increasing serum TG or TGAb levels, or patients have suspected diseased-related clinical symptoms. For patients preserving thyroid, neck ultrasound should be taken regularly and elevated TG above baseline should prompt further evaluation for recurrent disease. We also recommend the monitoring of MSO is at least 20 years since the median recurrence time in our study was 14 years and cases of late recurrence have been reported [9].

This study has several limitations. First, most cases from the literature review increased the heterogeneity of this study, weakening the validity. Second, follow-up time among the patients significantly varied, and in some of the patients it was not long enough, which may influence the exact outcomes. Third, surgeries being performed by surgeons in different institutions may also further impact the prognosis, even if the same surgical approaches were used. An ideal method would be conducting a prospective, randomized cohort to determine the optimal surgical option and examine the role of RAI in patients with MSO confined to the ovary. However, it is unrealistic due to its low feasibility. Last, we excluded many records mainly due to language and lack of institutional access. Further research is needed to determine optimal disease management.

## Conclusion

Patients with MSO confined to the ovary had an excellent survival outcome, despite varied treatment strategies, but the recurrent rate was relatively high. We recommend USO as the preferred surgical option for this population since more aggressive surgery does not improve outcomes and the benefits of RAI are uncertain.

## Supplementary Information

**Additional file 1:.** Database of our study

**Additional file 2:.** Univariate and multivariate analysis of RFS.

**Additional file 3:.** Univariate and multivariate analysis of OS

## Data Availability

All data generated or analyzed during this study are included in this published article and its supplementary information files. The datasets used and/or analyzed during the current study are available from the corresponding author upon reasonable request.

## Abbreviations

MSO: Malignant struma ovarii
PTC: Papillary thyroid carcinoma
FTC: Follicular thyroid carcinoma
FVPTC: Follicular variant of papillary thyroid carcinoma
USO: Unilateral salpingo-oophorectomy
BSO: Bilateral salpingo-oophorectomy
TAH/sTAH: Total/subtotal abdominal hysterectomy
TT: Total thyroidectomy
RAI: Radioiodine therapy
EBRT: External beam radiotherapy
rh-TSH: Recombinant human thyroid stimulating hormone
TERT: Telomerase reverse transcriptase
NA: Not applicable
