# Supplementary material for: Clinical characteristics and survival outcomes of malignant struma ovarii confined to the ovary
Source: BMC Cancer. 2021 Apr 9;21:383. doi: 10.1186/s12885-021-08118-7 (PMC8033663; doi:10.1186/s12885-021-08118-7)
Supplement: Supplementary file 2 — Additional file 2:. Univariate and multivariate analysis of RFS. [file 12885_2021_8118_MOESM2_ESM.docx]

**Table S2** Univariate and multivariate analysis of RFS

| Factors | N |  | Univariate analysis | | | |  | Multivariate cox regression analysis | | | | | | |
| --- | --- | --- | --- | --- | --- | --- | --- | --- | --- | --- | --- | --- | --- | --- |
|  |  | Mean survival(yrs) | | 10-year DSF rate | | p |  | OR | (95% CI) | | | | | p |
| Age (years) |  |  | |  | | 0.220 |  |  | |  | | | |  |
| >=55 | 30 | 12.5 | | 78.0% | |  |  |  | | | | | | |
| <55 | 94 | 13.2 | | 61.9% | |  |  |  | | | | | | |
| Ascites |  |  | |  | | 0.320 |  |  | | | | | | |
| Yes | 19 | 9.7 | | 46.3% | |  |  |  |  | | | | |  |
| No | 97 | 14.3 | | 67.0% | |  |  |  |  | | | | |  |
| Tumor size (cm) |  |  | |  | | 0.202 |  |  |  | | | | |  |
| >=8 | 27 | 13.7 | | 55.7% | |  |  |  | | |  |  | | |
| <8 | 45 | 20.4 | | 73.0% | |  |  |  | | | | | | |
| Follicular carcinoma subtype ^a^ |  |  | |  | | 0.141 |  |  | | |  |  | | |
| Yes | 23 | 10.0 | | 40.4% | |  |  | 1.612 | | | 0.705, 3.682 | 0.257 | | |
| No | 100 | 16.4 | | 76.0% | |  |  |  | | | | | | |
| Initial Surgical option |  |  |  | | >0.2/5 | |  |  | | | | | | |
| cystectomy | 10 | - | | 16.7% | |  |  |  | | | | | | |
| USO | 42 | - | | 61.0% | |  |  |  | | | | | | |
| BSO | 7 | - | | - | |  |  |  | | | | | | |
| TAH + BSO | 26 | - | | 56.7% | |  |  |  | | | | | | |
| Debulking | 22 | - | | 85.7% | |  |  |  | | | | | | |
| Initial RAI therapy ^a^ |  |  | |  | | 0.161 |  |  | | | | | | |
| Yes | 25 | 6.0 | | 94.4% | |  |  | 0.288 | | | 0.038, 2.172 | | 0.227 | |
| No | 93 | 12.9 | | 60.1% | |  |  |  | | | | | | |
| Initial Chemotherapy |  |  | |  | | 0.304 |  |  | | | | | | |
| Yes | 5 | 9.3 | | 53.3% | |  |  |  | | | | | | |
| No | 114 | 14.0 | | 64.7% | |  |  |  | | | | | | |

*a* Factors applied to multivariate analysis; * *p* < 0.05*

Abbreviations: RFS, recurrent-free survival; USO, unilateral salpingo-oophorectomy; BSO, bilateral salpingo-oophorectomy; TAH, total abdominal hysterectomy; RAI, radioiodine therapy.
