# Supplementary material for: Clinical characteristics and survival outcomes of malignant struma ovarii confined to the ovary
Source: BMC Cancer. 2021 Apr 9;21:383. doi: 10.1186/s12885-021-08118-7 (PMC8033663; doi:10.1186/s12885-021-08118-7)
Supplement: Supplementary file 3 — Additional file 3:. Univariate and multivariate analysis of OS [file 12885_2021_8118_MOESM3_ESM.docx]

**Table S3** Univariate and multivariate analysis of OS

| Factors | N |  | Univariate analysis | | | |  | Multivariate cox regression analysis | | | | | |
| --- | --- | --- | --- | --- | --- | --- | --- | --- | --- | --- | --- | --- | --- |
|  |  | Mean survival(yrs) | | 10-year OS rate | | p |  | OR | (95% CI) | | | | p |
| Age (years) |  |  | |  | | 0.240 |  |  | |  | | |  |
| >=55 | 31 | - | | - | |  |  |  | | | | | |
| <55 | 94 | - | | 86.0% | |  |  |  | | | | | |
| Ascites^a^ |  |  | |  | | 0.010* |  |  | | | | | |
| Yes | 19 | 15.3 | | 65.0% | |  |  | 5.807 | 0.970, 34.770 | | | | 0.054 |
| No | 98 | 35.8 | | 93.3% | |  |  |  |  | | | |  |
| Tumor size (cm) |  |  | |  | | 0.965 |  |  |  | | | |  |
| >=8 | 28 | 23.5 | | 93.3% | |  |  |  | |  |  | | |
| <8 | 45 | 23.3 | | 85.7% | |  |  |  | | | | | |
| Follicular carcinoma subtype |  |  | |  | | 0.952 |  |  | |  |  | | |
| Yes | 23 | 19.0 | | 85.7% | |  |  |  | |  |  | | |
| No | 100 | 38.6 | | 93.7% | |  |  |  | | | | | |
| Initial Surgical option |  |  |  | | >0.2/5 | |  |  | | | | | |
| cystectomy | 10 | - | | - | |  |  |  | | | | | |
| USO | 42 | - | | 85.5% | |  |  |  | | | | | |
| BSO | 7 | - | | - | |  |  |  | | | | | |
| TAH + BSO | 27 | - | | 75.6% | |  |  |  | | | | | |
| Debulking | 22 | - | | 85.7% | |  |  |  | | | | | |
| Initial RAI therapy |  |  | |  | | 0.480 |  |  | | | | | |
| Yes | 25 | - | | - | |  |  |  | |  |  | | |
| No | 94 | - | | 87.6% | |  |  |  | | | | | |
| Initial Chemotherapy |  |  | |  | | 0.525 |  |  | | | | | |
| Yes | 6 | - | | - | |  |  |  | | | | | |
| No | 114 | - | | 87.4% | |  |  |  | | | | | |
| Recurrence |  |  | |  | | 0.008* |  |  | | | | | |
| Yes | 27 | - | | 75.6% | |  |  | 99.268 | | 0.061, 162390 | | 0.223 | |
| No | 97 | - | | - | |  |  |  | | | | | |

*a* Factors applied to multivariate analysis; * *p* < 0.05*

Abbreviations: OS, overall survival; USO, unilateral salpingo-oophorectomy; BSO, bilateral salpingo-oophorectomy; TAH, total abdominal hysterectomy; RAI, radioiodine therapy.
